# Osteoblastoma of the rib with CT and MR imaging: a case report and literature review

**DOI:** 10.1186/1477-7819-10-49

**Published:** 2012-03-07

**Authors:** Jing Ye, Liqing Liu, Jingtao Wu, Shouan Wang

**Affiliations:** 1 Department of Medical Imaging, Yang Zhou University Academy of Clinical Medicine, Yangzhou 225001, China

**Keywords:** Bone neoplasm, Osteoblastoma, Computer Tomography (CT), Magnetic Resonance (MR)

## Abstract

Osteoblastoma is a rare bone tumor which is mostly found in the vertebral column and long bone. We describe a 59-year-old woman with osteoblastoma in the right fifth posterior segment of the rib, whose presenting symptoms were right back pain for two years and awakened at night. Chest computer tomography (CT) and thoracic spine magnetic resonance (MR) imaging findings included an expansile lesion of the right fifth rib and an ossified matrix. Surgical resection of the lesion confirmed a benign osteoblastoma. 12 months follow-up revealed disappearance of right back pain. Rib osteoblastoma in plain film has been described previously; however, to our knowledge this is the only case report emphasized in CT and MR imaging.

## Background

Osteoblastoma is an uncommon bone tumor, which accounts for approximately 1% of all bone tumors [1]. Osteoblastoma can occur in any part of the skeleton. The most common sites are the verterbral column (34%) and long bone (30%). The rib is involved in less than 5% of patients [2,3]. The lesion is most frequently observed in patients less than 30 years of age, with approximately 70% of all cases occurring in the second or third decade of life. Males are affected more often than females in a ratio of approximately 2 to 1. Symptoms and signs are variable, but the characteristic symptom is a localized, dull pain that does not get worse at night in most cases and cannot be relieved by salicylates [4]. Osteoblastoma is a highly vascular and locally aggressive tumor and needs to be well imaged for complete surgical removal. Rib osteoblastoma has been described previously; however, to our knowledge there has been no case report using computer tomography (CT) and magnetic resonance (MR) imaging of rib osteoblastoma. We present a case of osteoblastoma of the rib, with an emphasis on the CT and MR imaging features.

## Case presentation

### History

A 59-year-old woman was admitted to hospital with a two-year history of right back pain. The pain was durative and had increased steadily in recent few months. She often awoke at night with the pain. There was point tenderness in the region of complaint sometimes occurring with pain radiation to the trunk. The pain cannot be relieved by aspirin. There was no fever or sweat. There was no history of tuberculosis, malignancy or surgery except hypertension. Her young sister had a history of lung cancer; nothing else was remarkable in family history. The woman consented kindly to our case report.

### Radiologic findings

**Figure 1:**
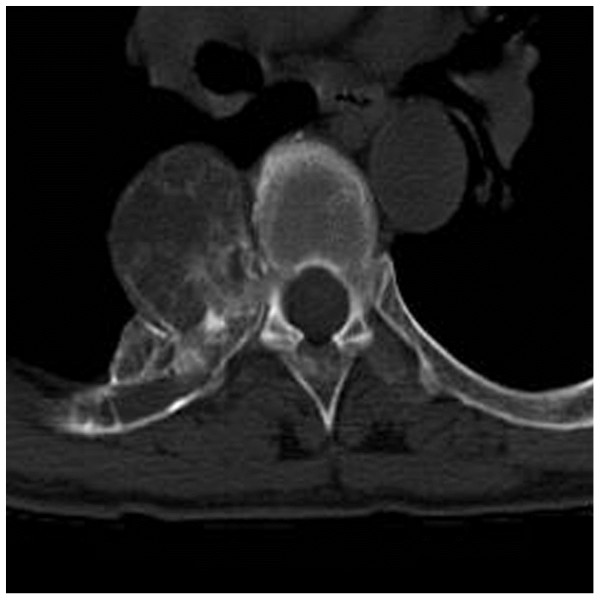
**CT reveals a lytic lesion in right fifth posterior rib with ossified matrix**.

**Figure 2:**
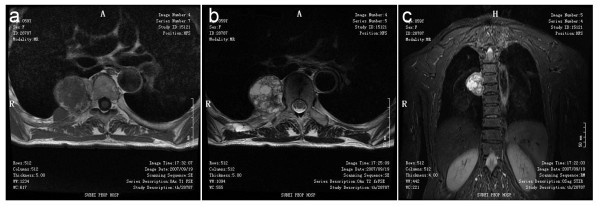
**MR finding in right fifth posterior rib**. MR imaging shows isointensity on a T1-weighted image (**a**), heterogeneous high signal pattern with a low signal rim on a T2-weighted image (**b**) and Coronal STIR imaging (**c**)

Non-contrast enhanced chest CT was obtained using a 16-channel multidetector scan. A paravertebral expansile lesion that involved the right fifth posterior segment of rib and protruded into the thoracic cavity was revealed. Cortical expansion and an ossified matrix were noted (see Figure 1). The mass was round in shape with a diameter of 5 cm × 4 cm, with clearly defined margins. MR imaging of the thoracic spine was performed. The mass had a relatively low signal in the T1-weighted image (T1WI). In the T2-weighted image (T2WI) and short-tau inversion-recovery (STIR) a heterogeneous signal pattern was revealed with a fine low signal intensity rim (see Figure 2A-C). According to radiologic findings and clinic history, the initial imaging impression was osteochondroma.

### Operative procedure and histopathology

The surgical approach consisted of a right posterior thoracotomy at the fifth intercostal space. After opening the pleural cavity, the superior, inferior and posterior margins of the tumor were clearly visible. The tumor revealed calcified areas mixed with cystic areas containing hemorrhage. A complete excision of the tumor was performed.

**Figure 3:**
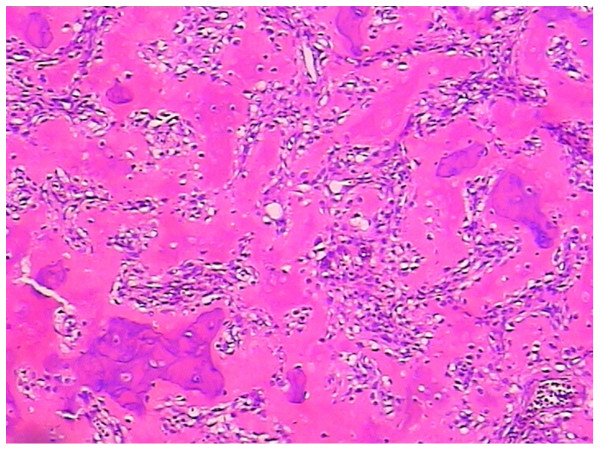
**(Hematoxylin and eosin staining, ×100) shows irregular woven bone and osteoid surrounded by activated osteoblasts**. Seven days after the operation the patient was discharged from hospital. At 12 months follow-up the preoperative symptoms disappeared completely and there were no post-operative complications.

Photomicrographs showed a bone-forming tumor with osteoblasts rimming osteoid and bony trabeculae. The tumor consisted of predominantly disorganized osteoid surrounded by numerous large osteoblasts within the vascular stoma. No mitosis or anaplasia were seen. These findings were compatible with benign osteoblastoma (Figures 3).

## Discussion

Osteoblastoma is an uncommon primary bone tumor. The most common sites are the verterbral column and long bone, followed by the feet, skull, clavicle and the ribs. The rib is involved in less than 5% of patients [2,3]. Patients are mostly less than 30 years of age, with approximately 70% of all cases occurring in the second or third decade of life, The woman in this case was 59 years old, and in a large report ages may range from 6 to 75 years. Males are affected more often than females in a ratio of approximately 2 to 1. Symptoms and signs of osteoblastoma are variable, but the characteristic symptom is a localized, dull pain that does not get worse at night in most cases and cannot be relieved by salicylates [4].

Histologically, osteoblastomas are benign primary bone tumors with some aggressive characteristics that can transform into osteosarcomas. Osteoblastomas will continue to grow and damage the bone and surrounding structures before excision, while the reoccurrence rate after operation is still 10% [5]. Therefore osteoblastomas should be well imaged before complete excision. Although X-ray is the most common imaging modality, CT and MRI are more useful for detecting small tumors and have superiority in displaying the size of the tumor and the relationship with adjacent tissues. What's more, CT can detect small mineralizations in the tumor, which can help diagnosis. MRI findings of osteoblastoma are not specific, with a low signal in T1-weighted images and a high signal in T2-weighted images. However, MRI can determine intraosseous and soft tissue extension more accurately.

Osteoblastoma may occur in an intramedullary, intracortical, or periosteal location. Therefore the radiological appearance of osteoblastomas are extremely variable and not specific [6,7]. Accurate preoperative diagnosis is often difficult and aggressive characteristics may be misinterpreted as signs of a malignant neoplasm. Radiologic findings of rib osteoblastoma have not been well documented because of its rarity. Kroon found that rib osteoblastomas had a central origin, but the imaging was not illustrated [4]; Gentry reported a periosteal rib osteoblastoma which implied a periosteal counterpart of osteoblastoma [8]. In our case study the rib osteoblastoma was ectogenic with expansion of the adjacent rib which signified a mixed growth pattern. According to radiologic findings of this case and previous literature we summarize that osteoblastoma, including rib osteoblastoma, may have three patterns of radiologic findings: a lesion > 2 cm in diameter with more prominent periosteal reaction; blow-out expansile behavior, similar to an aneurysmal bone cyst, with multiple central small calcifications, a thin shell of peripheral periosteal bone and a well-defined margin; and a more aggressive type with bone expansion and destruction, adjacent soft tissue infiltration and intermixed matrix calcification.

## Conclusion

We report an osteoblastoma of the rib, which was eosteolytic, expansile and well circumscribed with variable quantities of calcification or ossification areas. Certain typical CT and MRI features of osteoblastoma are well demonstrated, but the ectogenic growth with expansion of the adjacent rib is very rare which has not been previously described.

## Consent

Written informed consent was obtained from the patient for publication of the Case report and any accompanying images. A copy of the written consent is available for review by the Editor in Chief of this journal.

## Abbreviations

CT: Computer Tomography; MR: Magnetic Resonance; STIR: short-tau inversion-recovery

## Competing interests

The authors declare that they have no competing interests.

## Authors' contributions

JY carried out studies and participated in the Literature review. LL participated in the Clinical information collecting including follow-up. JW paticipated in the Literature review, and SW paticipated in imaging reading. All authors read and approved the final manuscript.
